# Photodynamic activation as a molecular switch to promote osteoblast cell differentiation via AP-1 activation

**DOI:** 10.1038/srep13114

**Published:** 2015-08-17

**Authors:** Toshihiro Kushibiki, Yupeng Tu, Adnan O. Abu-Yousif, Tayyaba Hasan

**Affiliations:** 1 Wellman Center for Photomedicine, Massachusetts General Hospital, Harvard Medical School, USA, 40 Blossom Street, Boston, MA 02114, USA; 2 Department of Medical Engineering, National Defense Medical College, Japan, 3-2 Namiki, Tokorozawa, Saitama 359-8513, Japan

## Abstract

In photodynamic therapy (PDT), cells are impregnated with a photosensitizing agent that is activated by light irradiation, thereby photochemically generating reactive oxygen species (ROS). The amounts of ROS produced depends on the PDT dose and the nature of the photosensitizer. Although high levels of ROS are cytotoxic, at physiological levels they play a key role as second messengers in cellular signaling pathways, pluripotency, and differentiation of stem cells. To investigate further the use of photochemically triggered manipulation of such pathways, we exposed mouse osteoblast precursor cells and rat primary mesenchymal stromal cells to low-dose PDT. Our results demonstrate that low-dose PDT can promote osteoblast differentiation via the activation of activator protein-1 (AP-1). Although PDT has been used primarily as an anti-cancer therapy, the use of light as a photochemical “molecular switch” to promote differentiation should expand the utility of this method in basic research and clinical applications.

Photodynamic therapy (PDT) has been approved for use in several countries for treatment of cancers and diseases associated with neovascularization^1^, including early-stage tumors and precancerous lesions in a wide range of tissues^2^. Multiple clinical studies of PDT, many of which were aimed at optimizing the conditions for its use, have been performed around the world.

In PDT, a photosensitizing compound is injected either systemically or directly into the lesion site. After the compound has accumulated in the tumor or other target tissue, the site is irradiated with laser light of a wavelength that can penetrate the surrounding tissue and activate the photosensitizing agent^3^. The PDT dose is the product of the photosensitizer concentration and the light dose delivered to the target and the PDT dose can impact the outcome in cells at a molecular level^4^. Photochemical activation of the compound results in production of reactive oxygen species (ROS)^5^. Specifically, irradiation excites the photosensitizer to an excited singlet state, which can experience one of two fates: decay to the ground state by fluorescence, which can be detected for imaging purposes, or electron spin conversion to the triplet state, which mediates the therapeutic effects of PDT. Triplet molecules can react with substrates to form radicals that interact with oxygen, resulting in macromolecular oxidation with potentially cytotoxic effects. Alternatively, the excited triplet state can transfer energy directly to molecular oxygen, forming ROS, the most cytotoxic products formed during PDT.

High levels of ROS can cause cell death, but ROS are also important mediators of intracellular signaling. Although detailed mechanism have yet to be unraveled, it is clear that ROS can target signaling at multiple levels, from the cell surface to the nucleus^6^. Receptor kinases and phosphatases, the upstream sensors of signaling pathways, are vulnerable to direct oxidation by ROS, which can block or otherwise modulate their physiological functions^7^. For example, growth-factor receptors are often activated by ligand-induced dimerization/oligomerization, leading to autophosphorylation of the cytoplasmic kinase domains^8^. Clustering and activation of such receptors in the absence of ligand have been demonstrated in response to UV light^9^, and this phenomenon is mediated by ROS^10^. Within the cytoplasm, cellular redox state influences the activity of several major pathways^11^ that control many aspects of cellular metabolism and proliferation via regulation of gene expression^12^. The transcription factors regulated by redox state include redox factor-1 (Ref-1), which mediates activation of activator protein-1 (AP-1) (Fos and Jun)^13^; nuclear factor kappa-B (NF-κB)^14^; p53^15^; hypoxia-inducible factor-1α (HIF-1α) and HIF-like factor^16^; and signal transducer and activator of transcription-3 (STAT-3)^17^.

A growing body of evidence suggests that ROS serve as important second messengers in pathways that regulate proliferation and differentiation. Oxygen tension influences proliferation and pluripotency of bone marrow–derived stem cells (BMSCs)^18^, in which ROS transduce a variety of signals related to control of differentiation^19^. Inhibition of the mitochondrial respiratory chain, which both alters the energy budget of the cell and induces ROS generation, enhances the pluripotency of human embryonic stem cells^20^. ROS also appear to be important for differentiation of stem cells into specific lineages, as well as activation of environmental responses in these cells. For example, ROS generation is required for differentiation of mesenchymal stem cells into adipocytes^21^ and for hypoxia-induced IL-6 production by ES cells^22^. Several studies have shown that ROS are critically involved in the differentiation of stem cells into the cardiac lineage, acting as second messengers in cardiomyocyte differentiation induced by both electrical stimuli^23^ and mechanical strain^24^; furthermore, ROS play a crucial role in the survival and proliferation of stem cell–derived cardiac cells^25^. Together, these observations demonstrate that ROS exert major influences on stem cell pluripotency and differentiation.

We hypothesized that PDT at doses lower than those conventionally used for cancer treatment might induce sufficient levels of ROS to influence differentiation without significant cell killing, thereby accelerating the differentiation of pluripotent cells. We tested this idea in osteoblast precursor cells and primary mesenchymal stromal cells, and observed that low-dose PDT indeed promoted osteoblast differentiation without significant cytotoxicity. We then investigated several signaling pathways potentially involved in osteoblast differentiation, and found that the AP-1 transcription factor is involved in mediating the effects of low-dose PDT by activation of Fos, Jun, and Fra transcripts.

## Results

### Evaluation of osteoblast differentiation after low-laser dose PDT

We investigated the effect of low–laser dose PDT on differentiation in the mouse osteoblast precursor cell line MC3T3-E1 and rat primary mesenchymal stromal cells. As a photosensitizer, we used 5-aminolevulinic acid (5-ALA), a biocompatible non-fluorescent heme precursor that induces synthesis and cellular accumulation of fluorescent protoporphyrin IX (PpIX)^26^. PDT is conventionally used to kill tumor cells; therefore, we first tested a range of light doses to establish optimal conditions for low-dose PDT that would not result in cell killing. Light doses of 1–3 J/cm^2^ caused no significant decrease in MC3T3-E1 cell viability 3 days after irradiation in the presence of 5-ALA (Supplementary Fig. S1), although a small but significant decrease in relative cell number was observed at 3 J/cm^2^ after 1 day. Laser energy densities above 4 J/cm^2^ resulted in more than 90% loss of cells viability.

**Figure 1:**
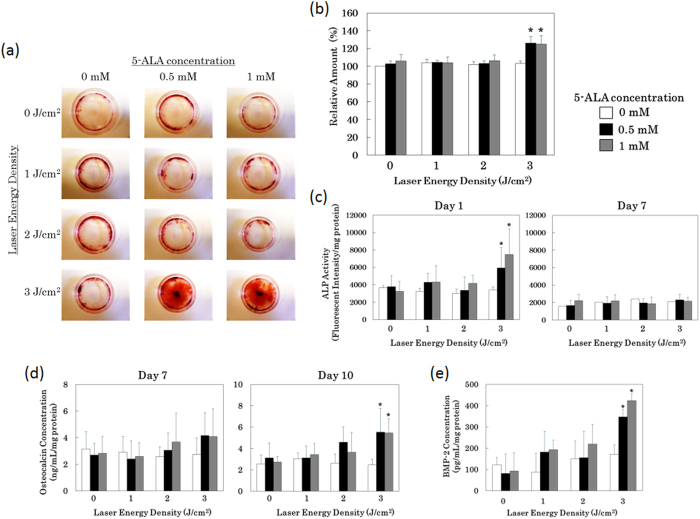
Elevated calcium deposition and induction of osteoblast differentiation markers after low-dose PDT. MC3T3-E1 osteoblast precursor cells were incubated for 3 hr with the indicated concentrations of 5-aminolevulinic acid (5-ALA), after which the drug was washed out and the cells were exposed to 0, 1, 2, or 3 J/cm^2^ of laser energy (30 mW/cm^2^; λ = 635 nm). After PDT, cells were switched to differentiation medium (10 nM dexamethasone, 10 mM β-glycerophosphate, 50 μg/ml ascorbic acid) and cultured for 7 days. (**a**) Culture dishes were subjected to Alizarin red staining to detect deposited calcium. (**b**) Deposited calcium was quantitated using the methylxylenol blue (MXB) method and normalized against the level in untreated cells. *p < 0.01 vs. 0 mM 5-ALA + 3 J/cm^2^. (**c**) Levels of secreted alkaline phosphatase (ALP) in the culture medium, an early differentiation marker, detected using a fluorescence-based assay. (**d**) Levels of osteocalcin in the culture medium, a late differentiation marker, detected by ELISA. (**e**) Levels of BMP-2 in the culture medium, an osteoblast marker, detected by ELISA. *p < 0.01 vs. 0 mM 5-ALA + 3 J/cm^2^.

After optimizing the conditions, we treated MC3T3-E1cells with low-dose PDT (light + 5-ALA) and examined the effect on calcium deposition as a surrogate marker of differentiation. Seven days after exposure to 3 J/cm^2^ in the presence of 5-ALA, MC3T3-E1 cells exhibited a significant increase in Alizarin red staining (Fig. 1a), which detects mineralized calcium. This effect was dependent on PDT dose: neither cells exposed to light in the absence of 5-ALA, nor cells exposed to lower doses of light in the presence of 5-ALA, exhibited elevated staining. Quantitation of the amount of deposited calcium using the methylxylenol blue (MXB) method revealed that cells exposed to low-dose PDT at 3 J/cm^2^ deposited 26% more calcium than untreated cells (Fig. 1b). We further characterized these cells by measuring the expression of osteoblast differentiation markers. A transient increase in expression of secreted alkaline phosphatase (ALP), an early differentiation marker was observed following exposure of cells to low-dose PDT (Fig. 1c). The late differentiation marker osteocalcin began to rise in cells exposed to low-dose PDT, increasing to 2-fold over control after 10 days (Fig. 1d). Likewise, BMP-2 levels were elevated 2-fold 7 days after treatment (Fig. 1e).

**Figure 2:**
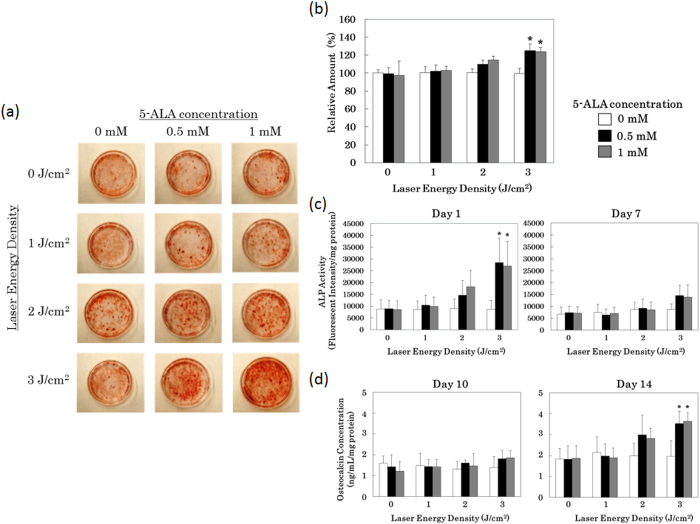
Low-dose PDT induces osteoblast differentiation in rat primary mesenchymal stromal cells. Rat primary mesenchymal stromal cells were incubated for 3 hr with the indicated concentrations of 5-aminolevulinic acid (5-ALA), after which the drug was washed out and the cells were exposed to 0, 1, 2, or 3 J/cm^2^ of laser energy (30 mW/cm^2^; λ = 635 nm). After PDT, cells were switched to differentiation medium (10 nM dexamethasone, 10 mM beta-glycerophosphate, 50 μg/ml ascorbic acid), and cultured for the indicated times. (**a**) Fourteen days after PDT, culture dishes were subjected to Alizarin red staining to detect deposited calcium. (**b**) Fourteen days after PDT, deposited calcium was quantitated using the methylxylenol blue (MXB) method. (**c**) Levels of secreted alkaline phosphatase (ALP) in the culture medium, an early differentiation marker, detected using a fluorescence-based assay. (**d**) Levels of osteocalcin in the culture medium, a late differentiation marker, detected by ELISA. *p < 0.01 vs. 0 mM 5-ALA + 3 J/cm^2^.

Inspired by our observations in osteoblasts, we performed similar experiments in primary rat mesenchymal stromal cells (MSCs) to determine if our findings can be generalized to other pluripotent stem cell types. As in the osteoblast precursor cells, low-dose PDT induced the MSCs to deposit calcium (Fig. 2a,b), express the early marker secreted ALP (Fig. 2c), and the late marker osteocalcin (Fig. 2d). Although the results were qualitatively similar in these two cell types, differentiation was slower in MSCs than in osteoblast precursors (e.g., compare Fig. 1d to Fig. 2d: osteoblast precursors expressed osteocalcin 10 days after PDT, whereas no significant increase in osteocalcin was observed in MSCs until day 14). Taken together, these data demonstrate that low-dose PDT induced osteoblast differentiation in osteoblast precursor cells and primary MSCs, as determined by both functional assays (calcium deposition) and expression of differentiation markers.

### AP-1 mediates induction of osteoblast differentiation after low-dose PDT

**Figure 3:**
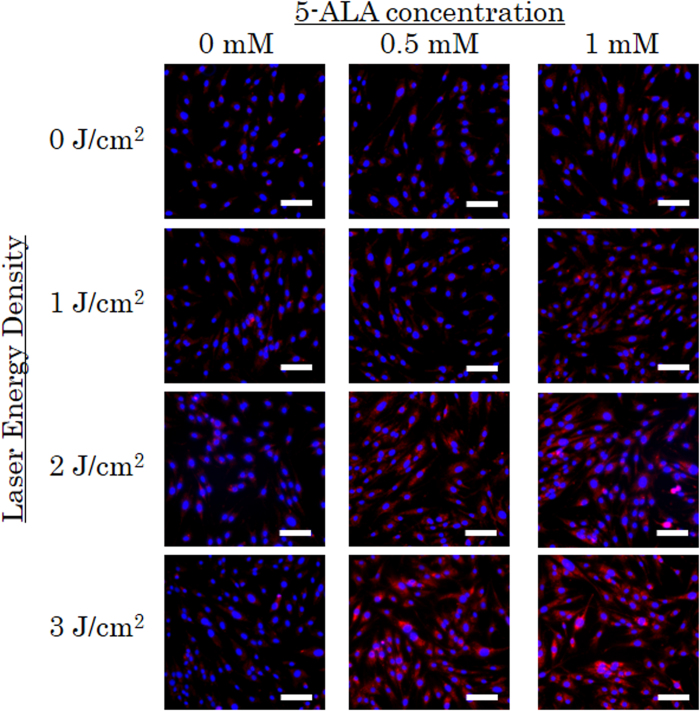
Intracellular ROS formation after low-dose PDT. MC3T3-E1 osteoblast precursor cells were incubated with CellROX reagent 30 min before low-dose PDT. CellROX reagent is non-fluorescent while in a reduced state and becomes fluorescent (red) upon oxidation by ROS. After low-dose PDT, cells were fixed and DAPI solution was added for the nuclear counterstain (blue). bar = 50 μm.

Next, we sought to identify the critical factors involved in the osteoblast differentiation we observed in MC3T3-E1 cells following low-dose PDT. Intracellular ROS imaging by using the fluorogenic probe revealed that intracellular ROS was enhanced by low-dose PDT at 3 J/cm^2^ (Fig. 3). We investigated several transcription factors regulated by intracellular ROS levels: activator protein-1 (AP-1), which regulates gene expression in response to a variety of conditions including cellular stress^27^; NF-κB, a major inducer of proinflammatory signaling^28^; HIF-1α, which mediates the response to hypoxia^29^; and STAT-3, which is involved in signaling in response to several cytokines and growth factor receptors^30^. Previous reports implicate each of these pathways in osteoblast differentiation. AP-1 promotes differentiation^31^,^32^, in part via protein–protein interactions with Runx2^33^ and Smads^34^. By contrast, NF-κB, HIF-1α, and STAT-3 have been reported to either stimulate or suppress differentiation depending on the context. NF-κB is activated under conditions that promote osteogenic differentiation of human adipose tissue derived stromal cells via a subsequent increase of transcriptional coactivator with PDZ-binding motif (TAZ) expression^35^, whereas in other systems inhibition of NF-κB leads to enhance Fra-1 expression in differentiated osteoblasts and is associated with increased bone matrix deposition and bone formation^36^. Hypoxia and other treatments that induce HIF-1α expression can elevate BMP-2 expression^37^ and promote calcium deposition^38^, whereas hypoxia is reported to inhibit osteogenic differentiation in some cell types^39^. Similarly, STAT-3 has been reported to both stimulate and inhibit osteogenesis in mesenchymal stem cells^40^. We elected not to investigate two other ROS-regulated factors, p53 and Nrf2, because these proteins are involved in pathways exclusively shown to suppress osteoblast differentiation^41^,^42^.

**Figure 4:**
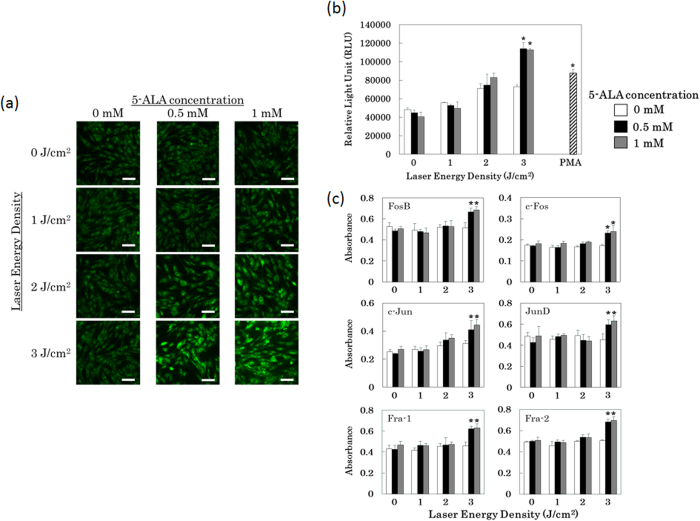
Activation of the AP-1 pathway following low-dose PDT. MC3T3-E1 osteoblast precursor cells were stably transfected with pGreenFire-AP1, a GFP/luciferase dual reporter of AP-1 pathway activity, and otherwise treated as described for Fig. 1. All data shown in (**a**)–(**c**) were collected 1 hr after PDT. (**a**) GFP signal produced by pGreenFire-AP1, detected by fluorescence microscopy. bar = 50 μm. (**b**) Quantitation of luciferase expressed from pGreenFire-AP1. PMA (positive control): cells were treated with 100 nM phorbol 13-myristate 12-acetate for 30 min to induce AP-1 transcription factor activity. Luciferase activities are expressed in graphs as relative luciferase units (RLU). *p < 0.01 vs. 0 mM 5-ALA + 3 J/cm^2^. (**c**) DNA binding levels of the indicated Fos and Jun family member subunits of AP-1 dimeric complexes. *p < 0.05 vs. 0 mM 5-ALA + 3 J/cm^2^.

In order to investigate AP-1 pathway activity, we stably transfected MC3T3-E1 cells with the pGreenFire-AP1 construct, which is activated by AP-1 transcription factor complexes, i.e., hetero- or homo-dimeric complexes of members of the proto-oncogene families Jun (c-Jun, JunB, JunD) and Fos (c-Fos, FosB). When activated, pGreenFire expresses both GFP and luciferase, enabling both fluorescence-microscopic detection via the GFP signal and quantitative transcription assays via the luciferase signal. Exposure of the transfected cells to low-dose PDT activated the AP-1 pathway, as reflected by GFP (Fig. 4a) and luciferase expression (Fig. 4b). These data revealed that AP-1-mediated signalling is activated in cells treated with 3 J/cm^2^ of laser energy. In addition, the DNA binding activity of multiple proteins of the Fos and Jun families were significantly upregulated in cells treated with 3 J/cm^2^ of laser energy in the presence of 5-ALA (Fig. 4c). The elevation DNA binding activity of AP-1, especially those previously reported to be involved in bone formation, indicates that the transcriptional activity of pGreenFire is likely to represent bona fide activation of the AP-1 pathway.

Protein kinase C (PKC) induces AP-1–dependent transcription^43^, whereas interference with PKC activity, by expression of kinase-dead or dominant-negative PKC^44^ or overexpression of a PKC-interacting protein^45^, abrogates AP-1 activation. To confirm that AP-1 activation was critical for osteoblast differentiation in PDT-treated cells, we exposed cells to PKC inhibitors and monitored the effect on osteoblast differentiation. Calcium deposition, as determined by Alizarin red staining, was significantly suppressed (Supplementary Fig. S2). Together, these findings demonstrate that the AP-1 pathway is activated by low-dose PDT, and that AP-1 activity is necessary for the induction of osteoblast differentiation following this treatment.

By contrast, none of the other factors we examined NF-κB, HIF-1α, and STAT-3 appeared to be involved in PDT-induced differentiation. In contrast to previous reports^46^, phosphorylation of NF-κB was not induced by low-dose PDT (Supplementary Fig. S3a), and an NF-κB promoter-reporter construct was not activated (Supplementary Fig. S3b). Consistent with this, and in sharp contrast to the PKC inhibitor experiments described above, the NF-κB inhibitor ammonium pyrrolidine dithiocarbamate had no effect on calcium deposition following low-dose PDT (Supplementary Fig. S3c). Likewise, HIF-1α was not expressed immediately after low dose PDT (Supplementary Fig. S4a), and the HIF-1α inhibitor 3-(2-(4-Adamantan-1-yl-phenoxy)-acetylamino)-4-hydroxybenzoic acid methyl ester had no effect on calcium deposition (Supplementary Fig. S4b). The levels of phosphorylated (i.e., activated) STAT-3 declined significantly following PDT, consistent with a previous report^47^ (Supplementary Fig. S5). These data suggest that these major transcription-factor pathways regulated by ROS are not involved in osteoblast differentiation induced by low-dose PDT.

## Discussion

The results of this study show that low-dose PDT promotes differentiation of osteoblast precursor cells and MSCs, as demonstrated by induction of calcium deposition and elevated expression of osteoblast differentiation markers (Figs 1 and 2). It is known that PpIX generated from exogeneous 5-ALA is localized mainly in the mitchondria initially. At later time point it diffuseds out into the cytosol and is also localized in cell membranes^48^. Intracellular ROS was enhanced by low-dose PDT at 3 J/cm^2^ (Fig. 3) and AP-1–dependent transcription was significantly induced (Fig. 4a and 4b), and the DNA binding activity of AP-1 components (FosB, c-Fos, c-Jun, JunD, Fra-1, Fra2) were upregulated (Fig. 4c), suggesting that this transcription factor plays an important role in the observed differentiation. Furthermore, inhibition of PKC suppressed calcium deposition in response to low-dose PDT (Supplementary Fig. S2), consistent with a requirement for AP-1 in this process. While direct AP-1 blocking was not tested, the well established relationship between PKC inhibition and AP-1 activation is consistent with our data.

**Figure 5:**
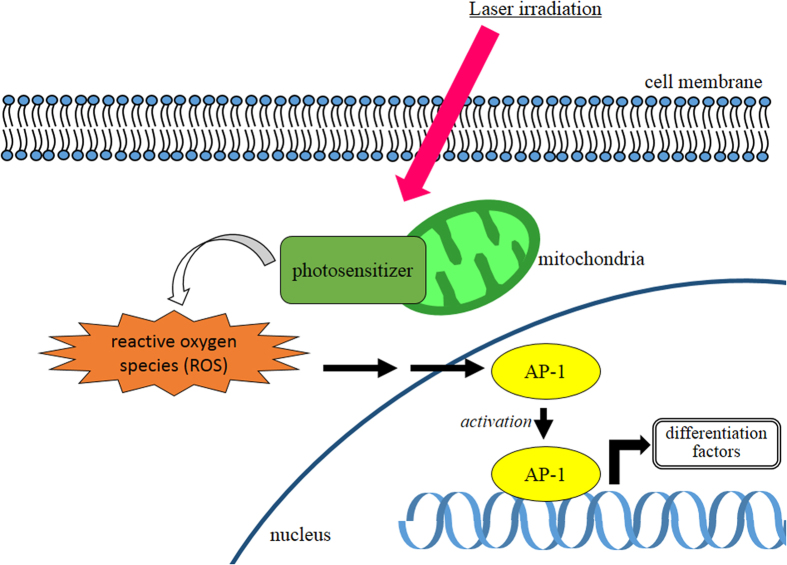
A proposed model for the osteoblast differentiation after low-dose PDT PDT activates AP-1 by enhancing the formation of ROS within cells. AP-1 exerts wide-ranging influences on cells differentiation.

The contribution of AP-1 to PDT-induced osteoblast differentiation is supported by several lines of evidence regarding the physiology and regulation of this transcription factor. First, AP-1 activity is upregulated by ROS^49^, possibly via stabilization of c-Fos and c-Jun transcripts or other post-transcriptional modifications^50^, and AP-1 activation is required for IL-10 production in response to PDT^51^. Although these previous studies focused on PDT regimens designed to kill tumor cells, a similar induction of AP-1 activity occurred at the sub-lethal doses used in our experiments. Second, AP-1 is responsive to oxidative stress^52^,^53^, suggesting that PDT may activate AP-1 by enhancing the formation of ROS within cells. Third, AP-1 exerts wide-ranging influences on pluripotency and differentiation. In addition to promoting the survival and growth of a variety of cell types^27^, it also promotes differentiation and controls lineage decisions in BMSCs^31^. In particular, AP-1 has been implicated specifically in osteoblast differentiation. Fra-1 and Fra-2, members of the Fos family, mediate bone matrix deposition and bone formation, and knockout mice lacking these proteins exhibit abnormal bone growth^54^,^55^,^56^. Furthermore, AP-1 interacts physically with the osteoblast-specific transcription factor Runx2^33^, and Fos and Jun family members are upregulated in response to TGF-β and BMP-2 in osteoblasts^32^. In light of these prior observations, our findings suggest that low-dose PDT enhances the formation of ROS, resulting in upregulation of AP-1 activity, which then proceeds to activate expression of bone markers and promote bone differentiation. Based on these experimental data, a suggested illustration depicting the role of AP-1 in cell differentiation after low-dose PDT is shown in Fig. 5. AP-1 is also reported as an important factor for regulating chondrocyte^57^ and adipocyte^58^ differentiation. Therefore, low-dose PDT-induced activation of AP-1 with the addition of appropriate differentiation medium to cells may regulate chondrocyte and adipocyte differentiation and warrant evaluation in subsequent studies.

Importantly, both HIF-1α and NF-κB were not activated by low-dose PDT, and their activity did not appear to be essential for promotion of osteoblast differentiation (Supplementary Figs. S3 and S4). Under conventional (high-dose) PDT, HIF-1α accumulates as a result of hypoxia, either due to consumption of molecular oxygen in the tumor cells or collateral damage to adjacent tumor vasculature^59^, although the latter effect is not observed *in vitro*.

To date, PDT has been used primarily to treat cancer and diseases associated with neovascularization. However, the results described here demonstrate that PDT could provide an effective means of changing cell fate by serving as a ‘molecular switch’ for regulatory proteins. Especially if the methods described here prove applicable to additional cell types, PDT-induced differentiation could be useful in multiple contexts. In basic studies of disease mechanisms and pathways, the ability to promote differentiation by exposing cells to light and small-molecule drugs would enable highly efficient and rapid production of desired cell types for use in experimental manipulations. Such an abundant and reliable source of differentiated cells could also be used to provide model cells for toxicity testing, novel target discovery, and drug-development studies. In the clinic, *in vitro* PDT-induced differentiation of precursor cells derived from patient tissues would decrease the waiting time required to obtain sufficient material for transplants or other procedures. PDT might even be used to promote *in situ* differentiation of precursor cells transplanted into diseased tissues or the patient’s own endogenous progenitor cells. Based on these potential uses, future studies should seek to demonstrate PDT-induced differentiation in an expanded range of cells, and to further characterize the molecular mechanisms underlying this phenomenon.

## Methods

### Cell culture

MC3T3-E1 cells (subclone 4, obtained from ATCC) were cultured in Minimum Essential Medium alpha (MEMα) without ascorbic acid (Life Technologies, Inc.) supplemented with 10% fetal calf serum (FCS, Thermo Scientific HyClone). Rat MSCs (obtained as described in the following paragraph) were cultured in Dulbecco’s Modified Eagle medium (DMEM, Life Technologies, Inc.) supplemented with 15% FCS. All media were supplemented with 100 units/mL penicillin and 0.1 mg/mL streptomycin. Cell culture was performed at 37 °C.

Rat MSCs were isolated from the bone shaft of femurs of 3-week-old male Fisher 344 rats as previously described^60^. Briefly, both the ends of rat femurs were cut away from the epiphysis, and the bone marrow was flushed out using a syringe (21-gauge needle) containing 1 ml of DMEM supplemented with 15% FCS and 50 U/ml penicillin/streptomycin. The cell suspension was placed into culture dishes. The medium was changed on the fourth day of culture and every 3 days thereafter. When the cells proliferated to subconfluence, usually after 7–10 days, they were detached by incubation for 5 min at 37 °C in phosphate-buffered saline (PBS, pH 7.4) containing 0.25% [w/v] trypsin/0.02% [w/v] EDTA. The cells were subcultured at a density of 2 × 10^4^ cells/cm^2^; subconfluent cells of the second passage were used for all experiments. Cell-surface markers of rat MSCs were confirmed by flow cytometry (CD29^+^, CD90^+^, CD34^−^, CD45^−^). All animal procedures were performed according to protocols approved by the Massachusetts General Hospital Subcommittee on Research Animal Care (2008N000159).

### Low-dose photodynamic therapy

Cells were seeded in 35-mm dishes (5 × 10^5^ cells/dish), cultured for 24 hr, and then cultured for 3 hr in 2 mL serum-free medium containing 0, 0.5, or 1.0 mM 5-ALA. After the 5-ALA incubation, cells were gently washed three times in PBS, and the incubation was replaced with 2 mL serum-free medium. Irradiation was performed using a 635-nm diode laser (Model 7401, High Power Devices, Inc) at 30 mW/cm^2^ for 0, 34, 67 or 102 sec (0, 1, 2 or 3 J/cm^2^) using a fiber-coupled illumination stage. After irradiation, the medium was replaced with 2 mL differentiation medium (complete medium containing 10 nM dexamethasone, 10 mM β-glycerophosphate, and 50 μg/ml ascorbic acid) and cultured for various times.

### Cell viability assays

After 1 day or 3 days of PDT, cells viability were quantitated by MTT assay, and then normalized against of cells viability in the untreated (0 mM 5-ALA + 0 J/cm^2^ from the same time point) sample on the same day.

### Alizarin red staining of calcium deposition

To evaluate calcium deposition, cells were rinsed three times with PBS and fixed with 4% formalin in PBS. Fixed cells were incubated with a 1% Alizarin red-S (Sigma–Aldrich, Co., MO) in aqueous solution (pH 6.5) for 15 min at room temperature, and then rinsed five times with PBS. Macro pictures of 35-mm dishes were taken by a digital camera (LUMIX, Panasonic Corporation, Japan).

### Quantification of calcium deposition

Cells were collected by scraping, centrifuged (100 *g*, 5 min), washed twice with PBS, and incubated overnight at 4 °C in hydrochloric acid solution. The calcium concentrations in the supernatants were determined by the methylxylenol blue (MXB) method using the Calcium E-test Wako (Wako Pure Chemical Industries, Osaka, Japan). After decalcification, the cells were washed three times with PBS and solubilized with 0.1% (w/v) SDS solution. The protein concentrations in solubilized cells were measured using the BCA Protein Assay Kit (PIERCE Biotechnology, Inc., IL). The calcium content of the cell layer was normalized to protein content; normalized concentrations are expressed as “/mg protein” in each graph.

### Protein detection

ALP activity in the culture medium was measured using the Alkaline Phosphatase Detection Kit, Fluorescence (Sigma–Aldrich, Co., MO). Other proteins were quantitated using commercially available ELISA kits, as follows: Osteocalcin in the culture medium, Mouse Osteocalcin EIA Kit (for MC3T3-E1 cells) and Rat Osteocalcin EIA Kit (for rat MSCs) (Biomedical Technologies, Inc., MA); BMP-2 in the culture medium, Human/Mouse/Rat BMP-2 Quantikine ELISA Kit (R&D Systems, Inc., MN); NF-κB p65, PathScan Phospho-NF-κB p65 ELISA kit (Cell Signaling Technology, Inc., MA); HIF-1α, Human/Mouse Total HIF-1α DuoSet IC (R&D Systems, Inc., MN); STAT-3, PathScan^®^ Phospho-Stat-3 (Tyr705) Sandwich ELISA Kit (Cell Signaling Technology, Inc., MA). For detection of NF-κB, HIF-1α, and STAT-3, cells were washed three times with PBS and lysed using the cell lysis buffer according to the manufacturer’s instructions. All protein concentrations was normalized to protein content of the cell layer using the BCA Protein Assay Kit (PIERCE Biotechnology, Inc., IL); normalized concentrations are expressed as “/mg protein” in each graph.

### ROS detection

Cells were seeded in 35-mm dishes (5 × 10^5^ cells/dish), cultured for 24 hr, and then cultured for 3 hr in 2 mL serum-free medium containing 0, 0.5, or 1.0 mM 5-ALA. CellROX reagent (Life Technologies, Inc.) at a final concentration of 5 μM was added to the cells and incubated for 30 minutes. After low-dose PDT, cells were fixed with 4% formaldehyde solution and DAPI solution (Life Technologies, Inc.) was added for the nuclear counterstain.The fluorescence imaging experiments were carried out on the Keyence BZ-9000 microscope using a 20× objective. Light and photomultiplier tube settings were consistent across experimental groups.

### AP-1 activity measurements

In order to investigate AP-1 pathway activity, pGreenFire-AP1-GFP-luciferase construct (System Biosciences, Inc., CA) was transfected into MC3T3-E1 cells using Lipofectamin 2000 (Life Technologies, Inc.). Seventy-two hours posttransfection, cells were seeded in 96-well plates and were selected for G418 (Sigma–Aldrich, Co., MO) resistance. After low-dose PDT to transfected cells, GFP fluorescence imaging was obtained by the Olympus FV1000 microscope using a 20× objective. Laser and photomultiplier tube settings were consistent across experimental groups. At the indicated times, supernatant was collected and luciferase activity was measured using the Bright-Glo Luciferase Assay System (Promega corporation, WI). Luciferase activities are expressed in graphs as relative luciferase units (RLU).

For the detection of DNA binding activity of AP-1 components (FosB, c-Fos, c-Jun, JunD, Fra-1, Fra2), AP-1 Family EZ-TFA Transcription Factor Assay Colorimetric (EMD Millipore, MA) was used. Cells were washed three times with PBS and lysed using the Nuclear Extraction Kit (EMD Millipore, MA). This assay kit detects specific transcription factor DNA binding activity in nuclear extract. It combines the principle of the electrophoresis mobility shift assay (EMSA) with the 96-well based enzyme-linked immune-sorbent assay (ELISA).

### Statistical analysis

Data represented as mean ± the standard derivation of the mean. Statistical significance (defined as *P* values of less than 0.01 or 0.05) was evaluated based on the unpaired Student’s *t* test (two-tailed). Values were determined in four or more independent experiments with samples collected in triplicate for each experiment.

## Additional Information

**How to cite this article**: Kushibiki, T. *et al*. Photodynamic activation as a molecular switch to promote osteoblast cell differentiation via AP-1 activation. *Sci. Rep*. **5**, 13114; doi: 10.1038/srep13114 (2015).

## Supplementary Material

Supplementary Information
