# Supplementary material for: Photodynamic activation as a molecular switch to promote osteoblast cell differentiation via AP-1 activation
Source: Sci Rep. 2015 Aug 17;5:13114. doi: 10.1038/srep13114 (PMC4538568; doi:10.1038/srep13114)
Supplement: Supplementary Information [file srep13114-s1.pdf]

Supplementary Information

Photodynamic activation as a molecular switch to promote osteoblast cell differentiation via AP-1 activation

Toshihiro Kushibiki[1,2], Yupeng Tu[1], Adnan O. Abu-Yousif[1], Tayyaba Hasan[1]

1. Wellman Center for Photomedicine, Massachusetts General Hospital, Harvard Medical School, USA
   40 Blossom Street, Boston, MA 02114, USA
2. Department of Medical Engineering, National Defense Medical College, Japan
   3-2 Namiki, Tokorozawa, Saitama 359-8513, Japan

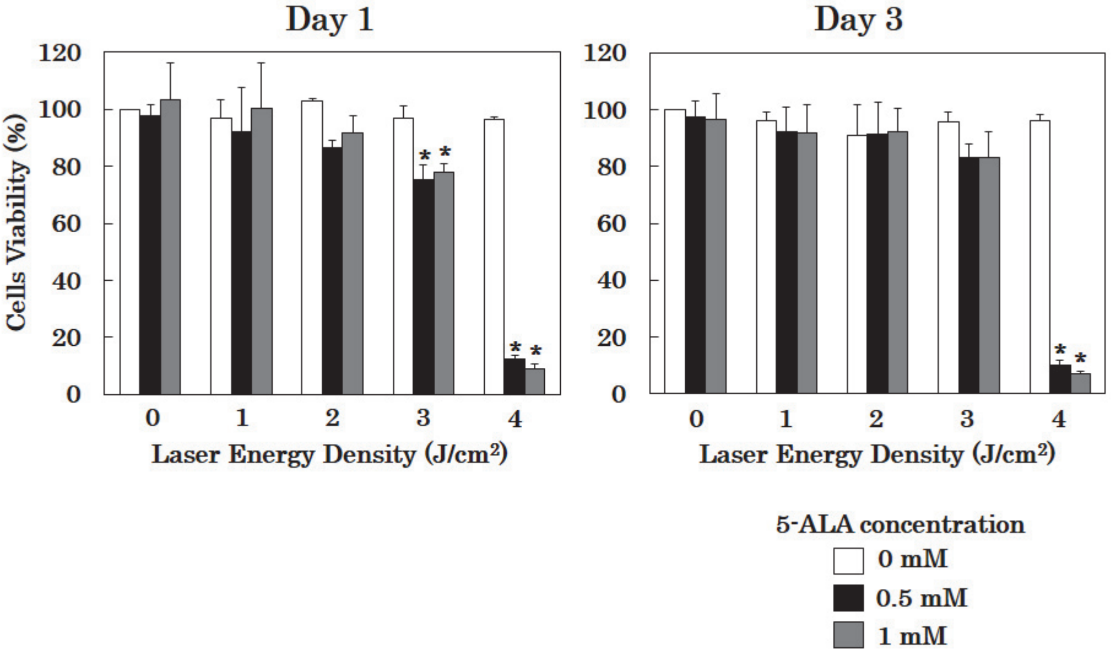

Supplementary Figure S1. Low-dose PDT does not cause cell killing

MC3T3-E1 osteoblast precursor cells were incubated for 3 hr with the indicated concentrations of 5-aminolevulinic acid (5-ALA), exposed to 0, 1, 2, 3, or 4 J/cm^2 of laser energy (30 mW/cm^2; wavelength=635 nm), and then switched to differentiation medium (10 nM dexamethasone, 10 mM beta-glycerophosphate, 50 µg/ml ascorbic acid) and cultured for the indicated times. After 1 day or 3 days, viable cells were quantitated by MTT assay, and then normalized against the number of viable cells in the untreated (0 mM 5-ALA, 0 J/cm^2) sample on the same day. After day 3, no cell killing was observed for doses ≤ 3 J/cm^2. At laser energy densities ≥ 4 J/cm^2, significant cell killing was observed. *, $p < 0.01$ vs. 0 mM 5-ALA + 3 J/cm^2.

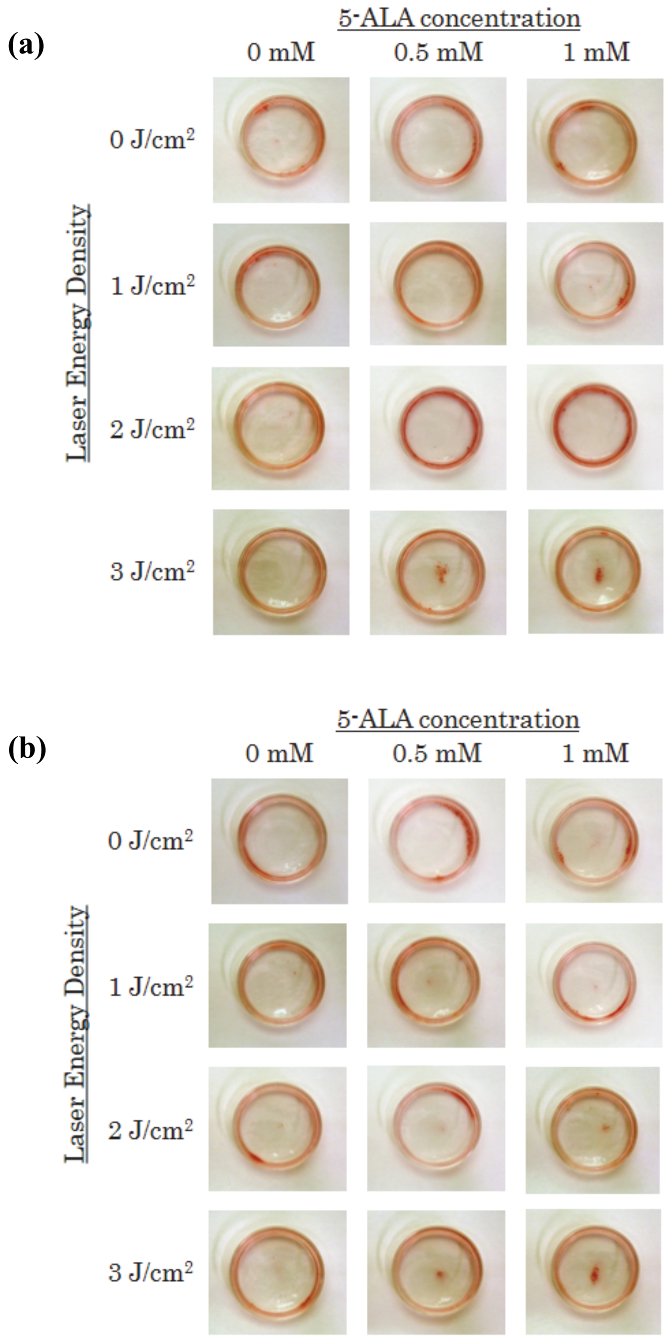

Supplementary Figure S2. Inhibition of PKC suppresses calcium deposition following low-dose PDT

Cells were treated as described for Figure 1, except that protein kinase C (PKC) inhibitors were added. Alizarin red staining was performed 7 days after low-dose PDT.

(**a**) Bisindolylmaleimide I (5 μM) was added 24 hr prior to PDT.

(**b**) Ro-32-0432 (100 nM) was added 24 hr prior to PDT.

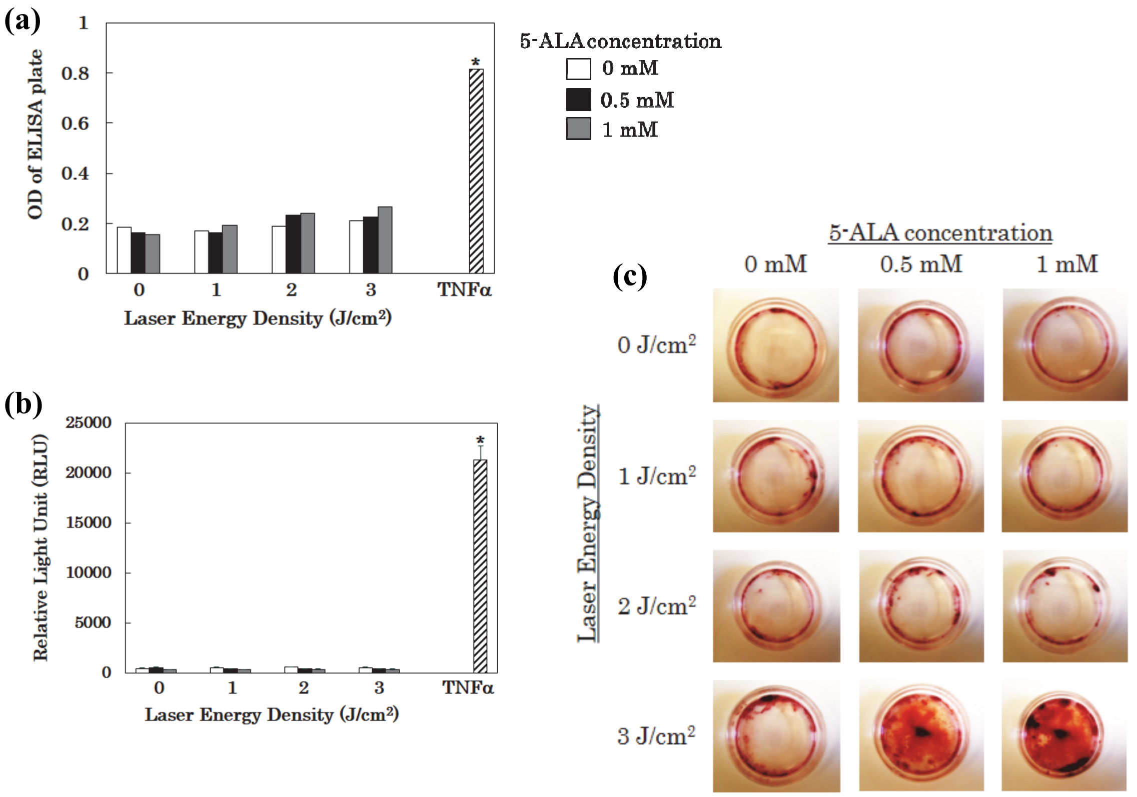

Supplementary Figure S3. NF-κB does not mediate differentiation induced by low-dose PDT

Cells were treated as described for Figure 1 except as indicated.

(**a**) Phospho-NF-κB p65 concentration of cell lysate 30 min after PDT, measured by ELISA. TNF-α (positive control): cells were treated for 10 min with 100 ng/mL tumor necrosis factor-α, an activator of the NF-κB pathway. *, $p < 0.01$ vs. 0 mM 5-ALA + 3 J/cm^2.

(**b**) Cells were transfected with a plasmid DNA vector containing a luciferase sequence under the control of the NF-κB enhancer element. One hour after PDT, luciferase activity (indicating NF-κB activation) was quantitated. TNF-α was used as a positive control, as in (**a**). *, $p < 0.01$ vs. 0 mM 5-ALA + 3 J/cm^2.

(**c**) One day before PDT, NF-κB inhibitor (APDC: ammonium pyrrolidine dithiocarbamate) was added to the medium at a concentration of 20 μg/mL. Culture dishes were subjected to Alizarin red staining 7 days after PDT.

(a)

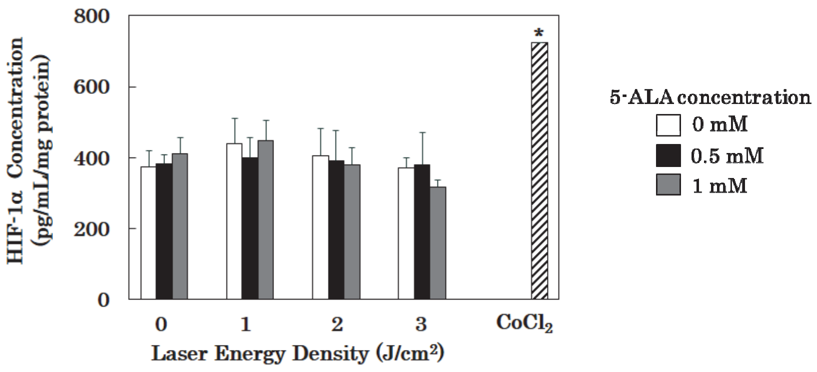

(b)

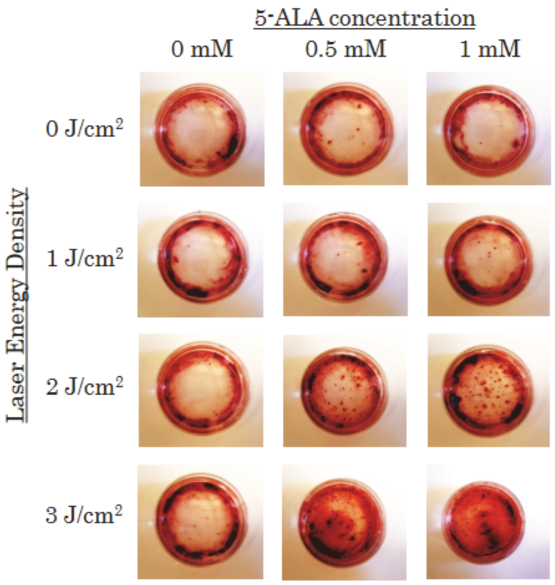

Supplementary Figure S4. HIF-1α does not mediate differentiation induced by low-dose PDT

(**a**) HIF-1α levels of cell lysate determined by ELISA immediately after PDT. $CoCl_2$ (positive control): Cobalt chloride, an activator of HIF-1α expression, was added at a concentration of 150 μM. *, $p < 0.01$ vs. 0 mM 5-ALA + 3 J/cm^2.

(**b**) One day before PDT, HIF-1α inhibitor [3-(2-(4-Adamantan-1-yl-phenoxy)-acetylamino)-4-hydroxybenzoic acid methyl ester] was added to the medium at a concentration of 2 μg/mL. Seven days after PDT, culture dishes were subjected to Alizarin red staining to detect deposited calcium.

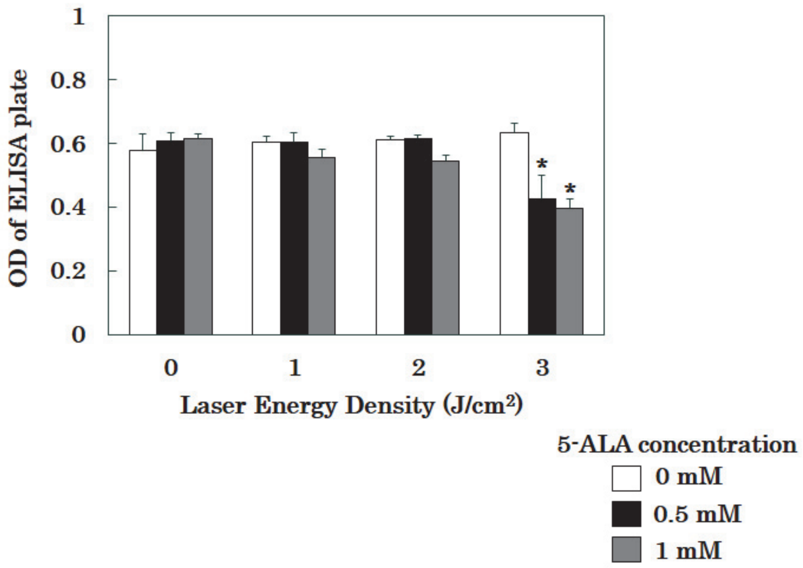

Supplementary Figure S5. STAT-3 activation is downregulated by low-dose PDT

Activated STAT-3 (phospho-Stat-3 Tyr705) of cell lysate was measured 1 hr after PDT by ELISA. *, $p < 0.01$ vs. 0 mM 5-ALA + 3 J/cm^2.
